# Periprosthetic Joint Infection Diagnosis: A Narrative Review

**DOI:** 10.3390/antibiotics12101485

**Published:** 2023-09-27

**Authors:** Konstantinos Tsikopoulos, Gabriele Meroni

**Affiliations:** 1 1st Department of Pharmacology, School of Medicine, Faculty of Health Sciences, Aristotle University of Thessaloniki, 54124 Thessaloniki, Greece; 2 One Health Unit, Department of Biomedical, Surgical and Dental Sciences, School of Medicine, University of Milan, 20133 Milan, Italy; gabriele.meroni@unimi.it

**Keywords:** prosthetic joint infection, diagnosis, molecular biology

## Abstract

Replacement of native joints aims to restore patients’ quality of life by relieving pain and improving joint function. While periprosthetic joint infection (PJI) affects a small percentage of patients, with an estimated incidence of 1–9% following primary total joint replacement, this postoperative complication necessitates a lengthy hospitalisation, extended antibiotic treatment and further surgery. It is highlighted that establishing the correct diagnosis of periprosthetic infections is critical in order for clinicians to avoid unnecessary treatments in patients with aseptic failure. Of note, the PJI diagnosis could not purely rely upon clinical manifestations given the fact that heterogeneity in host factors (e.g., age and comorbidities), variability in infection period, difference in anatomical location of the involved joint and discrepancies in pathogenicity/virulence of the causative organisms may confound the clinical picture. Furthermore, intra-operative contamination is considered to be the main culprit that can result in early or delayed infection, with the hematogenous spread being the most prevalent mode. To elaborate, early and hematogenous infections often start suddenly, whereas chronic late infections are induced by less virulent bacteria and tend to manifest in a more quiescent manner. Last but not least, viruses and fungal microorganisms exert a role in PJI pathogenesis.

## 1. Introduction

### 1.1. Role of Biofilm in PJIs

Biofilm formation is a principal feature in all types of prosthetic infections, including PJIs. In particular, biofilm has been consistently identified on various metal components, cement, bone, and fibrous tissue, and detached biofilm clumps have been recovered from joint fluid and diseased tissues [1,2]. Biofilm is typically defined as a complex microbial structure consisting of one or more species embedded in exopolysaccharides, proteins, teichoic acid, lipids, and extracellular DNA (eDNA) matrix. Its formation on prosthetic implants begins with bacterial adherence via surface features such as pili, fimbriae, flagella, and glycocalyx. Biofilm formation is partially regulated by quorum sensing, a communication system between bacteria dependent on population density [3]. Bacterial biofilms can be as thick as 100 µm, with biofilm-embedded bacteria being up to 1000 times more resistant to most antimicrobial agents compared to those in the planktonic form [4]. Numerous mechanisms have been described to account for the increase of antimicrobial resistance. These include the hindered or incomplete penetration of antimicrobial agents through the matrix, which has been demonstrated to neutralise and dilute their efficacy. Additionally, the reduced microorganism’s growth rate observed within biofilm regions results in antibiotics being ineffective for their action on cellular activity. Furthermore, the existence of efflux pumps contributes further to the development of antimicrobial resistance [5]. As a result, eradicating these slow-growing biofilms with antibiotic treatment alone is almost impossible. When standard sample and culture procedures are utilised, biofilm greatly complicates bacterial separation, thus decreasing the sensitivity of the microbiological analysis [6].

### 1.2. Microorganisms Implicated in PJIs

#### 1.2.1. Bacterial PJI

PJIs are typically classified as early-onset, delay-onset, or late-onset infections, with infections occurring within the first three months, between three and twelve to twenty-four months, or beyond twenty-four months, respectively [7]. Among bacterial species, 50% of PJI are supported by *Staphylococcus aureus*, *Staphylococcus epidermidis*, and other coagulase-negative staphylococci (CNS) [8]. Risk factors for *S. aureus* prosthetic joint infections are rheumatoid arthritis, prosthetic devices, diabetes, and *S. aureus* nasal colonisation [9]. Moreover, *S. aureus* has been linked to a much greater risk of failure when compared to other bacteria, with failure linked to early infection and the presence of bacteremia [10]. 

*S. epidermidis* strains are the leading cause of PJIs worldwide; however, their role as a pathogen remains controversial [11]. The isolation and differentiation of the other CNS species are variable and remain challenging in a laboratory to distinguish one species from another [12]. *Staphylococcus lugdunensis* is increasingly identified not only from PJIs but also from osteomyelitis, endocarditis, and bacteremia, which has received special attention in recent years [13,14]. *Staphylococcus capitis*, *Staphylococcus warneri*, *Staphylococcus hominis*, and *Staphylococcus haemolyticus* are other species found in PJIs [15]. 

*S. aureus* and CNS infections are sometimes worsened by the so-called “small colony variants (SCV)”, which are subpopulations of bacteria defined by unusual phenotypes and harmful features identified with varying frequency in PJIs [16]. SCVs had a sluggish growth rate, aberrant colony shape, odd metabolic characteristics, and even when clonally linked to co-isolated normal phenotypic bacteria, up to one-third displayed conflicting susceptibilities utilising E-test or disc diffusion tests [17,18]. SCVs are likely linked with recurrent or relapsing infections because they may penetrate and live inside non-professional phagocytes, sheltered from the host immune system and antimicrobial agents [19]. Nevertheless, no clinically significant difference has been demonstrated between individuals with PJI caused by SCVs and those with PJI caused by staphylococci with normal phenotypes [20].

Streptococci cause around 10–20% of PJIs, with total knee arthroplasties (TKA) and total hip arthroplasties (THA) as the most frequent source of isolation [21]. The most commonly identified streptococcal species are *Streptococcus agalactiae* (accounted for more than a third of streptococcal PJIs) and *Streptococcus dysgalactiae*, whereas viridans streptococci and *Streptococcus pneumoniae* are uncommon [22,23]. Comorbidities, *S. agalactiae* as the infecting organism, surgical care, and antibiotic therapy without rifampicin have all been linked to a worse result. For β-hemolytic streptococcal PJIs, current recommendations still prescribe 4- to 6-week treatment with penicillin G or ceftriaxone [21]. Diabetes, obesity and higher relapse are frequently connected with *S. agalactiae* infection [21]. Streptococci are more common in acute hematogenous infections, while enterococci in early postoperative infections [24].

Around 2–11% of PJIs are supported by Enterococci frequently associated with staphylococci (both CNS and CPS), gram negative bacilli (*Klebsiella* spp., *E. coli*, *Proteus* spp., *Pseudomonas aeruginosa*, *Morganella* spp., *Serratia marcescens*, *Enterobacter cloacae*, *Acinetobacter* spp.), anaerobes (*Finegoldia magna*, *Peptinophilus asaccharolyticus*, *Peptostreptococcus micros*, *Bacteroides fragilis*), and yeast (*Candida albicans*, *Candida parapsilosis*). Furthermore, polymicrobial infections accounted for almost half of all enterococcal PJIs with a high rate of treatment success, indicating that enterococcal PJIs are not difficult to treat [25,26]. In an investigation of 55 patients with enterococcal PJI, the most often isolated species was *Enterococcus faecalis*, followed by *E. faecium* and *E. casseliflavus*, while polymicrobial infections were widespread. There were 75 enterococcal PJI events, with hip 54.6%, knee 40%, elbow 3%, and shoulder 3% prosthesis involved [26].

Gram negative bacteria are isolated up to 23% among all PJIs, but isolation incidence in total knee and shoulder arthroplasties has been reported to be higher than 40% [27]. As found in a previous study, the frequency of multidrug-resistant gram negative arthroplasty infections increased from 5.3% in 2003 and 2004 to 8.1% in 2011 and 2012, with a comparable increase in identified strains such as *E. coli*, *K. pneumoniae*, *P. aeruginosa*, and *M. morganii* [28].

The prevalence of PJIs attributed to anaerobic bacteria ranges from 3% to 24%, contingent upon the affected joint and the timing of symptom manifestation. Among these anaerobic pathogens, *Propionibacterium acnes* (formerly *Cutibacterium acnes*) represents the most commonly encountered species [29]. The presence of *C. acnes* in tissue samples from PJIs has long been regarded as contamination, particularly if present in just a sample obtained from a patient [30]. PJI is now defined as the presence of bacteria in at least two perioperative tissue and/or synovial fluid samples [31]. Despite its low virulence, *C. acnes* has been related to various foreign body diseases, such as prosthetic valve endocarditis and meningitis caused by shunts and mechanical valves [32]. Immunosuppression, rheumatoid arthritis, and intra-abdominal pathology are all risk factors for clinical *Bacteroides* sp. infections [33]. Chin and colleagues reported an uncommon case of *B. fragilis* bacteremia and vertebral osteomyelitis in which the vertebral bodies were involved due to the continuous spread of polymicrobial disease through breaches in the gut mucosal barrier [34]. The translocation of some harmful bacteria from the gut was also described by Yeung, who presented a case report of a 61-year-old lady who had late PJI of a TKA supported by *B. fragilis* as a result of gut translocation mediated by temporary bacteremia and subsequent seeding of her TKA after a routine screening colonoscopy [35].

*Clostridium* species are most commonly detected in fractures with substantial wound environmental contamination during trauma while commonly identified in post-traumatic wounds; osteoarticular infection is uncommon [36]. The laboratory diagnosis of clostridial infection strictly depends on the collection and treatment of tissue. However, diagnosis can be complicated by the fact that bones and joints Clostridium species infections are frequently polymicrobial, with *Clostridium difficile* and *Clostridium perfringens* being the most common Clostridia isolated [37]. Although identification might be time-consuming, species other than *C. perfringens* exhibit diversity in sensitivity tests, including penicillin, cephalosporins, carbapenems, and clindamycin [38].

There is limited empirical data indicating that *Parvimonas micra* (formerly known as *Peptostreptococcus micros*), a pathogen found in the oral and gastrointestinal tract, is capable of causing periprosthetic joint infection. However, prior research has demonstrated that *P. micra* can lead to septic arthritis in elderly or immunocompromised individuals with tooth abscesses and periodontal disease, affecting native and prosthetic joints [39,40,41]. Randall et al. described the case of a healthy person (67-year-old female with a 2-year history of gingivitis) with recurrent gingivitis who developed a periprosthetic joint infection following total knee arthroplasty, highlighting that the full dental history should be obtained to reduce the risk of infection from oral pathogens such as *P. micra* [42]. In a case study, Ryan et al. documented a case of septic arthritis affecting the native hip joint caused by *P. micra* in a 65-year-old male patient with a preexisting diagnosis of coronary artery disease. According to the research, risk factors to develop *P. micra* PJI include crystal-induced arthritis, dental infection, concomitant pseudogout, and past oral infections [43].

Moving to the less common etiological agents, *Corynebacterium* species and *Finegoldia magna* (formerly *Peptostreptococcus magnus*) have been historically considered common findings in polymicrobial infections [44]. *Corynebacterium* spp. may be identified in up to 5% of PJIs and may be the only organism detected in 2% of cases. *C. striatum* is the most common species identified in PJI cases, while *C. jeikeium*, *C. amycolatum*, *C. tuberculostearicum* and *C. simulans* were less prevalent [45,46]. Recently, *Odoribacter splanchnicus* was described as a new uncommon anaerobes linked to a rare case of PJI in an 82-year-old woman diagnosed with hip infection [47].

As the number of prosthetic joint replacements grows, so does the risk of prosthetic joint infection, particularly in individuals exposed to animals. *Pasteurella multocida* is found in the nasopharynx or gastrointestinal tract of wild animals, cats, and dogs, so zoonotic infections induced by this bacterium are exceedingly uncommon (50% of dog bites and 75% of cat bites contain this bacterium in their saliva) [48]. Shih and Chen reported the case of a 52-year-old male with a liver transplantation followed by PJI due to cat scratches and bites [49]. Similarly, Ranavaya and Awadh described a total left knee replacement in a 75-year-old female with T2DM and hypertension. After an initial sepsis due to an abscessed cat bite (supported by *E. faecalis*) treated with ampicillin and doxycycline for 14 days, a second infection caused by *P. multocida* caused the prosthesis to be removed [50]. The isolation from the synovial fluid is rare, as found in the case report of Lafont and colleagues, who found this zoonotic agent in the knee of a 92-year-old woman [51].

Approximately 2% of all PJI cases are caused by mycobacterial microorganisms [52]. *Mycobacterium tuberculosis* is a rare cause of PJI, accounting for just seven instances (0.3%) recorded in one centre during 22 years [53]. However, several studies linked early hip/knee PJI with rapidly growing nontuberculous mycobacteria (NTM), suggesting these mycobacteria as a differential diagnosis in cases of early hip/knee PJI [52,54,55]. In the systematic review by Santoso and colleagues, the incidence of *M. tuberculosis* PJI was 43%, and the NTM accounted for *M. fortuitum* (22.6%), *M. abscessus* (8.6%), *M. chelonae* (6.9%), and *M. bovis* (6.9%), with an early infection (<3 months) in 25% of cases and a late (>3 months) in 75% [56]. Table 1 summarises the isolation frequencies of bacterial pathogens linked to PJIs.

#### 1.2.2. Fungal PJIs

Fungal microorganisms are rarely implicated in PJIs, with the estimated prevalence varying between 1% and 2% of cases [62]. In contrast to bacterial-induced PJI, symptoms following a fungal device-associated infection PJI tend to be subtle in nature [63]. It should be noted that the main culprits are *Candida* species in 80% of cases [64].

When it comes to diagnosing fungal PJIs, traditional enriched cultures still represent the main diagnostic tool. However, the major shortcoming of the above tool is the culture duration, as the minimum amount of incubation required for a reliable assessment is 4 weeks [65]. Therefore, the need for adopting new techniques such as next-generation sequencing (NGS) and/or PCR is advisable as they can be potentially used as adjuncts to enhance the diagnostic accuracy of the existing methods.

For the management of fungal-induced PJIs, adopting an MDT approach is highly recommended, as a significant number of patients with fungal infections are immunocompromised. In more detail, the members of the team should be as follows: revision arthroplasty orthopaedic surgeons, clinical microbiologists with relevant experience, physiotherapists, plastic surgeons, dieticians, nurse practitioners, and vascular surgeons. On top of that, it has been evidenced that early collaboration between multiple subspecialties yields promising outcomes for PJI patients [66]. For most patients, surgical intervention is required, with two-stage revision being the most appropriate treatment for chronic cases [66]. Regarding antifungal chemotherapy, it should be noted that the duration of treatment should be long enough and supplemented with antibiotics for bacterial superinfection coverage [63]. 

#### 1.2.3. Impact of Viral Infections

While bacterial pathogens have traditionally been the primary culprits behind PJIs, emerging evidence suggests that viral infections might be influential in exacerbating the risk and severity of these complications. It is worthy of note that viruses may induce moderate to severe arthritis and/or osteitis. Furthermore, viral infections pose a significant risk factor for surgical site infection, with rheumatological patients at a higher risk for developing more severe and long-lasting diseases [67].

The human immunodeficiency virus (HIV) is a highly significant viral agent within this particular environment. The presence of HIV infection has been correlated with immunosuppression, potentially compromising the host’s immune response against various infections, including those that may arise in the context of joint prostheses. Research findings indicate that HIV-positive individuals have a higher susceptibility to developing PJIs than the community. The immunosuppressive condition resulting from HIV infection diminishes the efficacy of the immune response, hence facilitating the colonisation of microorganisms in the vicinity of prosthetic joints and the subsequent development of infections [68,69].

The herpesviridae family, which encompasses herpes simplex virus (HSV) and varicella-zoster virus (VZV), has also attracted considerable interest due to its probable involvement in PJIs. These viruses can form latent infections within nerve cells and subsequently reawaken under conditions of stress or immunosuppression. The reactivation of these viruses can potentially induce localised inflammation around prosthetic joints, establishing a favourable bacterial colonisation environment. Furthermore, the inflammatory response triggered by the recurrence of a viral infection could potentially undermine the integrity of the implant and, hence, lead to its failure [70,71].

The impact of viral infections on PJIs extends beyond immunosuppression and inflammation. Studies indicate that there exists a potential for direct interaction between specific viruses and bacterial pathogens, hence exerting an influence on the microbial ecology in the vicinity of prosthetic joints. Viruses have the capacity to modify the composition of the microbiome, potentially leading to the proliferation of harmful microorganisms. The modification of the microbiome has the potential to elevate the susceptibility to bacterial colonisation and subsequent infection [72].

The processes by which viruses exert an effect on PJIs are multifaceted and complex, encompassing immunosuppression and alterations in microbial ecology. The ongoing progress in medical science has the potential to elucidate the intricate relationships between viral infections and PJIs, which could result in enhanced prevention measures, diagnostics, and treatment modalities. Consequently, this advancement may ultimately improve outcomes and quality of life for individuals who have undergone prosthetic joint implantation.

### 1.3. Clinical Picture and Diagnostic Criteria

The clinical picture of PJI patients varies significantly depending on whether the condition is acute or chronic. For the former, immediate postoperative pain with or without wound healing issues is often present [73]. In addition, wound dehiscence and surgical site drainage are frequently encountered in this group of patients. Regarding fever, the presence of this sign yields high specificity but low sensitivity; therefore, the diagnosis cannot be relied upon in the absence of it [74].

On the other hand, in the setting of chronic PJI, the clinical presentation tends to be more indolent. To elaborate, clinical examination may be inconclusive on that occasion, and sometimes orthopaedic surgeons fail to clinically distinguish between aseptic failures and chronic periprosthetic joint infections [73].

PJI diagnosis requires a precise definition. The Musculoskeletal Infection Society (MSIS) and the Infectious Diseases Society standardised PJI criteria in 2011, thus improving diagnostic confidence and research cooperation. In 2018, new diagnostic criteria were published to overcome the limits of consensus-based definitions [75]. The 2018 method with new diagnostic tests has 97.7% sensitivity and 99.5% specificity, compared to 86.9% and 79.3% for the 2011 MSIS criteria (Table 2) [76]. The diagnostic criteria for PJI in the 2011 MSIS classification system include two major (primary) and six minor (secondary) criteria. Patients must meet either one major or four minor criteria to be diagnosed with PJI [77]. With the new 2018 MSIS criteria, the score-based system classifies as infected a patient that meets at least one major criteria or collects a total score ≥6 with minor criteria or inconclusive pre-op score or dry tap [77]. The real difference between the two systems (except for the use of specific biomarkers) is based on the moment of the diagnosis that in the 2018 MSIS criteria can be done in the pre-operative and intraoperative settings.

### 1.4. Laboratory Markers: Serum and Synovial Fluid

Laboratory tools are important for diagnosing PJI; their primary goal is to distinguish between infection and other reasons for implant failure. The diagnosis is focused on a combination of clinical findings and laboratory tests based on various samples (e.g., peripheral blood, histological tissues, microbiological cultures, Erythrocyte Sedimentation Rate (ESR), as well as intraoperative findings targeting different biomarkers (e.g., D-dimer, C-Reactive Protein (CRP), synovial leukocyte esterase, and synovial alpha-defensin) [75] (all the tests are summarised at the end of this paragraph in Table 3). 

Synovial and serum biomarkers are biological indicators used to diagnose PJI. These biomarkers have a crucial role in developing PJI, and their concentration is higher in PJI patients. CRP, ESR, interleukin-6, procalcitonin (PCT), D-dimer, tumor necrosis factor-alpha, intercellular adhesion molecule-1 (ICAM-1), and lipopolysaccharide binding protein (LBP) are biomarkers found in serum. 

D-dimers are a kind of fibrin degradation product generated by plasmin action on the fibrin clot, resulting in its dissolution. Some investigations have demonstrated that both systemic and local infections can induce fibrinolytic activity, hence causing elevated levels of D-dimer [79]. D-dimer testing has shown potential in the early detection of postoperative infection. However, it is important to acknowledge several limitations of this diagnostic method, primarily its lack of specificity. Additionally, it is worth noting that increased levels of D-dimer may not always indicate the presence of infection, as they might also indicate an unrelated inflammatory condition [80]. In recent studies, researchers have shown that D-dimer has potential as a serological marker for diagnostic purposes in periprosthetic joint infection (PJI), with a sensitivity of 89% and specificity of 93% [80,81].

The Musculoskeletal Infection Society (MSIS) poses CRP and ESR together as a minor criterion for diagnosing PJI. CRP is an acute-phase protein produced in the liver after inflammation. CRP levels are often higher, although they can also be elevated due to postoperative inflammation and other diseases. Earlier antibiotic therapy may also reduce CRP levels [82]. Focusing on PJI diagnosis, its sensitivity and specificity have been reported to vary in different studies, primarily depending on the cut-off value used for illness diagnosis. In the meta-analysis by Berbari and colleagues, sensitivity and specificity were 97% (95% CI, 93–99%) and 91% (95% CI, 87–94%), respectively [83]. ESR rises owing to an increase in fibrinogen and other normal plasma proteins, as well as the creation of aberrant circulating proteins from necrotic tissue; for this reason, it is considered a nonspecific marker of PJI (if evaluated alone). ESR is combined with CRP to enhance the accuracy and precision of diagnosing PJI. The study conducted by Sigmund et al. revealed that the sensitivity of CRP and ESR when employing modified MSIS criteria was found to be 70.6% and 40.6%, respectively. However, when these two measures were used in conjunction, the sensitivity increased to 75%. [84].

Interleukin-6 (IL-6) is a cytokine monocytes and macrophages release during inflammation, causing liver cells to create CRP. The thyroid gland primarily synthesises PCT (a precursor of calcitonin). However, in cases of inflammation, infection, lipopolysaccharide, microbial toxins, and other inflammatory mediators induce the production of PCT from various other cells in the body, including white blood cells, the spleen, the kidney, and the liver [85]. Both IL-6 and PCT can be easily measured in both serum and synovial fluid; however, Yoon and colleagues advised that PCT should not be used as a diagnostic marker for localised infections but only for systemic bacterial infections [86].

Interleukin 1β (IL-1β) is a major proinflammatory cytokine produced by several cell types, such as macrophages and monocytes. It is synthesised due to microbes, other cytokines, antigen-presenting cells, and immune complexes. It acts as a stimulant for the liver to create acute-phase proteins and plays a crucial role as a pyrogen [87]. Research has indicated that the sensitivity of synovial IL-1β ranges from 66.7% to 100%, while its specificity ranges from 87% to 100% [88,89,90].

Several biomarkers found in synovial fluid, such as inflammatory proteins, cytokines, and antimicrobial peptides (including alpha-defensins, Human neutrophil elastase 2, bactericidal/permeability protein, neutrophil gelatinase-associated lipocalin, and lactoferrin), have been investigated as potential diagnostic indicators for prosthetic joint infection (PJI).

Alpha-defensins are a group of cationic peptides that are released by neutrophils in response to various types of microbes [91]. The regulation of their production is governed by pro-inflammatory cytokines such as IL-1β, IL-6, and TNF-α. Based on the established MSIS criteria, widely regarded as the benchmark, the diagnostic performance of alpha-defensins in synovial fluid for detecting PJI demonstrated a sensitivity and specificity of 100%. ELISA and lateral flow assay are used to detect α-defensins [92].

The Leucocyte Esterase (LE) strip test identifies neutrophil-secreted Leucocyte Esterase enzyme linked to the concentration of neutrophils in synovial fluid, representing PJI. In terms of sensitivity and specificity, Alpha-defensins have demonstrated more promising and trustworthy findings than the Leucocyte esterase strip test. The presence of blood in synovial negatively affects the leucocyte esterase strip test but does not affect the results of an alpha-defensins test [93].

Calprotectin (CPT) is a protein that binds to calcium and zinc and is mostly found in the cytoplasm of neutrophils. Upon activation of neutrophils, it is released into the extracellular environment, demonstrating antimicrobial properties [94]. In preliminary prospective research conducted by Wouthuyzen-Bakker et al., a comparison was made between 19 patients diagnosed with PJI and 42 control. The findings indicated that CPT may indicate PJI when the concentration exceeds 50 mg/L. The observed cut-off level demonstrated a high diagnostic accuracy, as shown by an area under the curve (AUC) value of 0.94. Furthermore, the sensitivity and the specificity were 89 and 90%, respectively [94].

**Table 3:** Serum and synovial fluid biomarkers.

	Biomarker	Origin	Cut-Off (Chronic)	Sensitivity (%)	Specificity (%)	PLR ^†^	NLR ^†^	Ref.
Serum	WBC count	WBC	5.48–10.5 × 10^9^ cells/L	21–42	89–94	3.3	0.63	[95]
	CRP	Hepatocytes	10 mg/L	84–95	85–97	3.9	0.23	[96]
	ESR ^1^	Erythrocytes	30 mm/h	100	54.7	3.5	0.27	[97]
	IL-6	Inflammatory cells	>6.6–12 pg/mL	62–100	85–100	5.1	017	[77]
	D-dimer	Dissolved fibrin clot	850 ng/mL	89	93	3.0	0.31	[80,81]
	PCT	Thyroid	0.1–0.75 ng/mL	91	13	5.6	0.48	[98]
Synovial	%PMNs	PMN	80%	90.7	87.8	7.1	0.14	[75]
	WBC count	WBC	>3000 cells/μL	41.6	89.7	8.7	0.14	[75,99]
	alpha-defensin	Neutrophils	3 mg/L	97	97	21.3	0.1	[100,101]
	Leukocyte Esterase	Neutrophils	2+ ^2^	93.3	77	-	-	[102]
	CRP	Hepatocytes	>6.9 mg/L	88	93	12.0	0.14	[77]
	IL-1β	Macrophages	312.7 pg/mL	97.3	94.6	10.6	0.13	[103]
	CPT	Neutrophils and macrophages		95	95	20.1	0.05	[104]
	TNFα	Macrophages		76	81	4.1	0.29	[104]

PLR = Positive Likelihood Ratio; NLR = Negative Likelihood Ratio; ^†^ the values are taken from [104]; ^1^ calculated in combination with CRP; ^2^ cut-off calculated using strips.

### 1.5. Microbiological Diagnosis of PJI: Culture-Based Methods

With a sensitivity varying from 65% to 95%, periprosthetic tissue samples give reliable specimens for identifying infecting microorganism(s) [105,106]. Currently, the minimum number of samples to be collected for reliable microbiological diagnosis is debated, but clinicians are concordant about at least three to five periprosthetic specimens from the tissue surrounding the implant should be collected [107,108]; a lesser number may cause misinterpretation, whereas a larger generally results in additional costs for the laboratory. For the pre-operative microbiological diagnosis of PJI, synovial fluid is typically the only sample available because it allows for the early identification of the pathogenic organisms and the evaluation of antibiotic resistance to develop the right therapeutic plan [109]. However, knee aspiration is a reasonably easy technique. On the contrary, hip aspiration usually necessitates ultrasonographic guidance. Moreover, in certain circumstances, hip aspiration does not give a significant volume of liquid for further analysis, necessitating injection and aspiration of normal saline into the joint [110]. The use of synovial fluid for the research of aerobic and anaerobic bacteria is included in the most widely available diagnostic algorithms [75,99,111]. However, pre-operative synovial fluid culture has limited sensitivity in chronic infections and cannot be relied on to rule out PJI [112]. Blood cultures should always be taken to detect a probable bloodstream infection in case of fever or chills [105,109].

Arthroscopic biopsy remains an appealing choice due to reduced soft tissue injury, little invasiveness, quick recovery, and improved view of the synovial membrane and components [113,114].

Periprosthetic tissue cultures had higher diagnostic sensitivity and specificity than synovial fluid aspirations. On the other hand, the frequencies of culture-negative PJI range from 7 to 12% [115]. As with synovial aspiration culture, it has been suggested to discontinue antibiotic therapy for two weeks before obtaining culture. Pre-operative antibiotics, culture methods, and bacteriostatic chemicals have all been linked to culture-negative infections PJI [115]. High-yield locations include the intramedullary canal and the prosthesis-bone junction. Samples should be collected using separate tools and quickly transferred to sterile transport bottles without coming into touch with drapes or gloves [99]. A single positive culture remains a diagnostic dilemma and should be considered with other results when reaching a diagnosis [75]. When virulent or unusual pollutants are identified, it suggests an underlying illness. Infection cannot be verified when common pollutants are identified, but further tests should be conducted [99].

The presence of a sinus communicating with the implant is regarded as a major criterion in the MSIS guidelines. Nonetheless, while some clinicians consider sinus canal swabs a viable material for PJI diagnosis, their usage is discouraged. In actuality, only a 50% concordance between sinus and deep cultures has been documented, with deep cultures having a higher chance of finding polymicrobial diseases due to contamination isolation [116]. 

Swabs are not suggested for sampling periprosthetic tissue or synovial fluid and should be avoided. Potential concerns connected with using swabs include increased contamination risk, lower specimen volume for culture, and pathogen growth suppression [117]. 

A two-week incubation period is frequently advised for diagnosing PJI, particularly in chronic PJI cultures [118]. Some researchers have hypothesised that culture plates may become contaminated during sampling or due to an extended incubation time [119]. Nevertheless, adhering to some fundamental microbiological recommendations, such as executing the procedures under sterile conditions, may keep contamination manageable, even if the plates are incubated for up to two weeks [106]. It is feasible, however, to shorten the incubation period by inoculating blood culture bottles with sonication fluid [120,121]. The reading of the plates implicates the identification of several colonial phenotypes, such as morphotypes and SCVs [122,123]. Surprisingly, when bacteria are cultured through culture media, most SCVs return to a normal phenotype [124]. SCVs can remain intracellularly and are less resistant to drugs than their wild-type counterparts, resulting in latent or recurring infections [19]. Two or more positive periprosthetic cultures and a single positive tissue culture with a very pathogenic microbe are regarded as clear proof of PJI. If, on the other hand, a low virulence bacterium develops only in one tissue culture, it should be considered in the context of other available information, such as sonication fluid culture [99,109].

Nevertheless, it is worth noting that a significant proportion of patients, ranging from 20% to 50%, exhibit negative cultures while presenting with evident clinical and laboratory indications of PJI. This scenario poses a diagnostic challenge sometimes called “culture-negative PJI” [115,125,126]. The “culture-negative PJI” frequency exhibits significant variability, which may be attributed to the different diagnostic criteria, microbiological culture methodologies, laboratory contamination rates, and patient cohorts included in the research [127,128]. 

The aetiology of “culture-negative PJI” may be categorised into three main scenarios: (i) patient-related factors, (ii) organism-related factors, and (iii) laboratory-related factors. Several patient characteristics have been identified as being linked with “culture-negative PJI”; these include elderly, smoking, and the occurrence of postoperative wound drainage [129]. A prevalent cause of negative aspiration cultures might be the use of local anaesthesia in the joint during the surgery since it possesses bacteriostatic properties [130]. Nevertheless, the primary factor contributing to the inability to identify pathogenic organisms using culture techniques is pre-operative antibiotic therapy [131,132]. Several authors [108,133,134] have hypothesised that a longer antimicrobial suspension time may increase the isolation of some difficult-to-grow microbes. According to research, 43% of “culture-negative PJI” are caused by mycobacteria, 46% by fungi, and 11% by *C. acnes*, *L. monocytogenes*, *Brucella*, *Coxiella burnetii*, and other uncommon organisms that do not grow on normal culture media [129]. Laboratory-related factors that may lead to false-negative culture findings include sample techniques, specimen transportation, growth media, and incubation duration [135]. For instance, samples may be improperly acquired from noninfected areas, and unintended transit delays or exposure to severe temperatures may result in tissue hydration and viability loss [135].

### 1.6. Molecular Diagnosis of PJI: Non-Culture-Based Methods

Polymerase chain reaction techniques (PCR) and NGS are examples of non-culture-based methodologies. PCR is a powerful diagnostic technique for PJI that may be used on various specimens, including tissue samples, synovial fluid, and sonicated prosthetic fluid [136]. Because the sensitivity and rapidity of this technique outperform those of tissue cultures, it is used in characterising bacterial colonies [137]. The PCR target can be amplified for a single organism or many microorganisms, giving rise to multiplex-PCR (mPCR). These systems’ ability to amplify different targets in a single process simplifies the approach, saves time and money, and shortens diagnostic times [138,139]. Many pharmaceutical companies have launched mPCR techniques, including SeptiFast by Roche, Genotype by Hain, Xpert by Cephaid, and Filmarray by Biofire. mPCR may detect and diagnose culture-negative PJI while also characterising the gene profile of bacteria through database comparison [140].

Broad-range PCR targeting the 16S rRNA gene has a sensitivity of 50% to 92% and a specificity of 65% to 94%, remaining still vulnerable to mistake; hence, the lack of sensitivity may result in a false-positive result. On the other hand, all bacteria can be recognised in a polymicrobial infection, lowering turnaround time [141,142,143]. When confronted with a scenario involving diverse cultural backgrounds, broad-range PCR can identify the prevailing bacterial strain at the site of infection. Non-viable bacteria may be recognised by PCR in a small sample volume and within a few hours [144], as well as in cultures from patients undergoing antibiotic therapy. PCR, in particular, can be used to provide culture-independent diagnostics. In cases where patients had received antibiotics during the two weeks before the surgery, the use of a pathogen panel for PJIs revealed that PCR of sonicated fluid exhibited a higher level of sensitivity compared to culture (88% vs. 70%). Conversely, no statistically significant alterations were seen in individuals who did not receive antibiotic therapy [145]. The inability to discriminate between living and dead bacteria and DNA contamination are the primary limitations of PCR-based diagnosis, resulting in a false positive result [146]. All pathogens, including previously unknown ones, may be detected using universal primers that can amplify bacterial or fungal DNA [147,148,149]. In order to detect the presence of *Kingella kingae*, an important pathogen in paediatric osteoarticular infections, it is necessary to perform a pathogen-specific PCR targeting the 16S gene [141].

Besides the important diagnostic role of PCR and mPCR, NGS has become increasingly essential in PJI diagnosis in recent years. This strategy frees laboratory operations from a culture-based approach and increases protocol sensitivity; however, the increased sensitivity is offset by a loss in specificity, resulting in likely false positive outcomes [150]. Culture-negative infections account for around 7–12% of all PJI; the use of metagenomic NGS leads to the detection of novel possible pathogenic bacterium in 16–44% of cases; an NGS can detect new pathogens in 4–67% of culture-positive cases [151]. NGS is typically classified into two types: targeted NGS and shotgun metagenomic. Targeted NGS, often referred to as 16S rRNA gene-based NGS shotgun metagenomics allows for the assessment of bacterial variety and the identification of microbial abundance. Because it is found in all bacteria, the 16S rRNA gene has become the most often utilised region for bacterial identification. As a result, instead of sequencing the complete genome to identify the pathogen, one specific gene may be sequenced. This method is undoubtedly less expensive and easier to comprehend than shotgun metagenomic sequencing. This approach, however, has certain drawbacks, including the inability to detect antibiotic resistance genes or virulence factors, the possibility of creating false negatives, and a certain bias in the quantification of each species [152,153]. On the other hand, the shotgun metagenomic technique identifies and quantifies all of the DNA in a sample, which can then be compared to reference genome databases to identify diseases. This technique may also be utilised straight from samples to diagnose unknown or even unculturable or unviable infections [140,154].

### 1.7. Artificial Intelligence-Based Diagnosis of PJIs

Natural language processing (NLP) technologies are rapidly being employed for clinical and research reasons, and they provide a means of effectively extracting data items inherent in the unstructured text of electronic health records [155,156,157]. The NLP method included three major components: text processing (sentence segmentation, assertion identification, and temporal extraction), basis extraction (knowledge-driven annotation), and classification [158].

## 2. Conclusions

When caring for PJI, determining the bacterium that caused the infection is essential to determine the proper antibiotics, long-term treatment, and prognosis for these patients. Historically, this was accomplished by the culture of synovial fluid before surgery and through the collection of tissue samples during surgery. In the context of PJI, these diagnostic approaches have been demonstrated to have a low sensitivity; hence, new diagnostic methods have been created to enhance the detection of microorganisms (Table 4).

It is undeniable that the diagnosis of PJI remains a challenging task for clinicians, given the lack of tests that can reliably rule out the presence of infection. By and large, implementing a stepwise approach is advisable when it comes to establishing the diagnosis of PJI. Last but not least, this review raises awareness about the impact of viruses and fungal microorganisms in relation to PJI.

## Figures and Tables

**Table 1:** Bacterial pathogens isolated from PJI cases.

Microorganism	Prevalence (%)	Reference
**Aerobic Gram-positive bacteria**		
*S. aureus* and *S. epidermidis*	50	[8]
*S. lugdunensis*	10–19.8	[15,57]
*S. capitis*	11	[15]
*S. warneri*	8	
*S. hominis* and *S. haemolyticus*	3	
Streptococci	10–20	[21]
*S. dysgalactiae*	41	[58]
*S. mitis/oralis*	36	
*S. anginosus*	14	
*S. agalactiae* and *S. pneumoniae*	4.5	
Enterococci	2–11	[25,26]
*E. faecalis*	89	[59]
*E. faecium*	9	
*Granulicatella adiacens* and *Kocuria rhizophila*	0.19	[60]
Aerobic Gram-negative bacteria		
*E. coli*	21.7	[61]
*Proteus* sp.	2.5	[60]
*Enterobacter* sp.	2.5	
*S. marcescens*	1.4	
*K. pneumoniae*	2.04	
*M. morganii*	1.9	[24]
*P. aeruginosa*	20.9	[61]
Anaerobic Gram-positive bacteria		
*Cutibacterium acnes*	4.5	[24]

**Table 2:** Comparison between the 2011 and 2018 MSIS criteria for diagnosis of PJI [75,78].

2011 MSIS Criteria	2018 MSIS Criteria
Major Criteria (at Least 1)	Major Criteria
Formation of a sinus tract communicating with the prosthesis; Isolation of the pathogen by culture from 2 different tissues or fluid samples obtained from the same affected joint.	Sinus tract connecting with joint or prosthetic visualisation; Two positive cultures of the same organism.
Minor criteria (at least 4)	Minor criteria→(score)
	Pre-operative diagnosis: ▪ ≥6 = infected ▪ 2–5 = probably infected ▪ 0–1 = not infected
Elevated CRP or ESR; Elevated synovial WBC count; Elevated synovial PMN%; Purulent material in the affected joint; Isolation of one microorganism in one culture tissue or fluid; Histologic study of periprosthetic tissue at ×400 magnification showed more than five neutrophils per high-power field in five high-power fields.	Elevated serum CRP or D.dimer→(2); Elevated serum ESR→(1); Elevated synovial WBC or LE→(3); Positive alpha defensin→(3); Elevated synovial PMN%→(2); Elevated synovial CRP→(1)
	Inconclusive pre-operative diagnosis or dry tap: ▪ ≥6 = infected ▪ 4–5 = inconclusive ▪ ≤3 = not infected
	Pre-operative score→(-); Positive histology→(3); Positive purulence→(3); Single positive culture→(2)

For abbreviations, refer to the next paragraph and Table 3.

**Table 4:** Available tests used to diagnose PJI.

Test	Diagnostic Criteria	Specificity (%)		Sensitivity (%)	Limitations	Ref.
Histology	>5 PMN per high-powered field in 5 separate microscopic fields	90		82	▪ reading variation among different pathologists ▪ alteration after antibiotic administration	[159]
Microbiological cultures	Organism identification	Blood cultures	100	85	▪ alteration after antibiotic administration ▪ contamination ▪ incubation time	[160]
		Container	99	26		
		Tissue sample	99	32		
PCR	Organism identification	74–100		50–96	▪ contamination	[161]
16-S targeting PCR	Organism identification	65–94		50–92		[162]
NGS	Organism identification	89.9		60.9		[163]

## Data Availability

All data presented in this study are available from the corresponding author upon request.
